# Mapping, intensities and future prediction of land use/land cover dynamics using google earth engine and CA- artificial neural network model

**DOI:** 10.1371/journal.pone.0288694

**Published:** 2023-07-24

**Authors:** Maysoon A. A. Osman, Elfatih M. Abdel-Rahman, Joshua Orungo Onono, Lydia A. Olaka, Muna M. Elhag, Marian Adan, Henri E. Z. Tonnang

**Affiliations:** 1 Department of Earth and Climate Sciences, Faculty of Science and Technology, University of Nairobi, Nairobi, Kenya; 2 International Centre of Insect Physiology and Ecology (icipe), Nairobi, Kenya; 3 Department of Forestry and Environment, Faculty of Forest Sciences and Technology, University of Gezira, Wad Madani, Sudan; 4 Department of Public Health, Pharmacology and Toxicology, University of Nairobi, Nairobi, Kenya; 5 Water Management and Irrigation Institute, University of Gezira, Wad Madani, Sudan; University 20 Aout 1955 skikda, Algeria, ALGERIA

## Abstract

Mapping of land use/ land cover (LULC) dynamics has gained significant attention in the past decades. This is due to the role played by LULC change in assessing climate, various ecosystem functions, natural resource activities and livelihoods in general. In Gedaref landscape of Eastern Sudan, there is limited or no knowledge of LULC structure and size, degree of change, transition, intensity and future outlook. Therefore, the aims of the current study were to (1) evaluate LULC changes in the Gedaref state, Sudan for the past thirty years (1988–2018) using Landsat imageries and the random forest classifier, (2) determine the underlying dynamics that caused the changes in the landscape structure using intensity analysis, and (3) predict future LULC outlook for the years 2028 and 2048 using cellular automata-artificial neural network (CA-ANN). The results exhibited drastic LULC dynamics driven mainly by cropland and settlement expansions, which increased by 13.92% and 319.61%, respectively, between 1988 and 2018. In contrast, forest and grassland declined by 56.47% and 56.23%, respectively. Moreover, the study shows that the gains in cropland coverage in Gedaref state over the studied period were at the expense of grassland and forest acreage, whereas the gains in settlements partially targeted cropland. Future LULC predictions showed a slight increase in cropland area from 89.59% to 90.43% and a considerable decrease in forest area (0.47% to 0.41%) between 2018 and 2048. Our findings provide reliable information on LULC patterns in Gedaref region that could be used for designing land use and environmental conservation frameworks for monitoring crop produce and grassland condition. In addition, the result could help in managing other natural resources and mitigating landscape fragmentation and degradation.

## Introduction

Land use/ land cover (LULC) dynamics have been an important research topic since the 1970s, due to its impacts and strong links to global, regional, and local climate variability and change [1–4]. Changes in LULC refer to the environmental changes as the result of anthropogenic activities and/ or natural consequences [5–7]. These changes are crucial in the modifications of micro-climate, bio(geo)diversity, ecosystem services, ecological and hydrological cycles and Earth’s biotic processes that cause adverse effects on socio-economic and sustainable livelihood aspects [6, 8, 9]. Hence, understanding LULC dynamics provide a vital factor for developing strategies for monitoring, evaluating, and conserving natural resources that are required for sustainable development [10, 11].

In Africa, LULC has been highly affected by severe and recurrent droughts, anthropic/human activities, and armed conflicts, among others [12, 13]. Particularly, the sub-Saharan Africa (SSA) region is projected to be highly susceptible to the effects of LULC changes, where many parts of the region experienced a diverse pattern of LULC dynamics, with significant transformations of forest and grassland into cropland [14, 15]. In addition, high levels of poverty, harvesting of fuelwood, charcoal production, agricultural expansion, settlements, unfavourable climatic events and land degradation in various agro-ecological zones are considered to be the major contributors to LULC changes in SSA [2, 16, 17]. Therefore, more research on the location, extent, magnitude and rate of LULC dynamics is still needed in SSA, where population is growing rapidly, coexisting with soil infertility and overuse of nature-based resources such as forests and water [2].

In Sudan, the natural resources are continuously diminishing, where forests and natural woodlands are lost as agricultural land expands [18]. Also, about 80% of the energy used in Sudan is produced from biomass, i.e., crop residues, charcoal and fuelwood [19]. These activities have primarily reformed LULC structure, especially in the main agricultural areas like Gedaref state in the past decades. Gedaref state is the primary rainfed crop production region in Sudan, where 80% of its population is engaged in farming [20]. However, in the last few decades, this area has been exposed to large-scale land degradation indicated by reduced vegetation coverage, and loss of soil fertility, among others [21]. This is basically due to unsuccessful land-use policies and practices used, such as sorghum mono‐cropping system and inappropriate methods of soil preparation and conservation [21–23]. Additionally, the expansion of rainfed mechanized agricultural schemes in Gedaref has played a significant role in LULC changes, which resulted in land degradation, environmental deterioration, and decline in agricultural productivity [24]. As a consequence, livelihood in this region has been highly affected. For instance, many pastoralists have lost their livestock or are forced to abandon livestock-rearing activities due to the loss of a considerable proportion of the traditional grazing lands [24]. Therefore, mapping LULC changes in Gedaref state could enable quantification of trends in agriculture, grassland, forest cover, and freshwater resources. This can help in managing agro-natural systems and improving land use policies [25].

Detection of LULC changes using satellite remote sensing is one of the approaches that has been intensively used to assess and understand various land-use dynamic forces at various spatio-temporal scales [26]. Despite the recent advancement in remote sensing and geospatial tools, there are inconsistency and lack of standards in LULC mapping and detection products, particularly at global and regional scales [27]. At a local scale, several studies have successfully assessed the dynamics of LULC and their drivers, as previously mentioned. Most of these studies have utilized commercial geospatial analytical tools like ArcGIS, Environment for Visualising Images (ENVI) and Earth Resources Data Analysis System (ERDAS), among others, for mapping and detecting LULC changes. However, in resource-limited countries like Sudan, such analytical tools might not be feasibly used. Also, these tools require computers with high-performance capability to analyze ’big’ satellite data. That also comes with time and cost implications in many developing countries. Moreover, most commercial geospatial tools do not allow automation of satellite data acquisition, processing and analysis. To overcome these challenges, cloud-based remote sensing and geospatial analytical tools like Google Earth Engine (GEE) have recently been introduced as freely available platforms for providing terabytes of images and advanced machine learning and artificial intelligence analytical tools (e.g., random forest (RF)) [28]. This could allow the development of relatively accurate semi- or fully automated LULC change detection approaches.

Furthermore, accurate LULC change layers could be efficiently used to predict future LULC patterns, which are also helpful for forecasting the vulnerability of ecosystems to, for instance, climate change. This requires a different set of tools that use artificial intelligence to simulate and mimic such future LULC dynamics. One of these tools is the cellular automata (CA) model, which has a high potential to effectively perform nonlinear spatially complex LULC change processes [29]. Cellular automata is a valuable approach for understanding LULC dynamics and their integral systems, especially when combined with other machine learning techniques, such as artificial neural network (ANN) [30, 31]. The CA-ANN is an artificial intelligence algorithm commonly used for simulating LULC change patterns, and it works on what-if scenarios [31, 32]. In spite of the complexity of LULC set up in any ecosystem, the CA-ANN model provides comparatively accurate future predictions that could deliver to stakeholders and policymakers of future LULC outlooks for informed planning [29, 33, 34].

Thus far, in Sudan, studies have mainly utilized the maximum likelihood (ML) classifier with Landsat and Advanced Spaceborne Thermal Emission and Reflection Radiometer (ASTER) datasets to characterize LULC dynamics in rainfed agricultural areas. Subsequently, LULC changes in these areas were estimated using different methods. For instance, in the Northern Kordofan region, LULC change was assessed between the periods 1973–2001 [35, 36] and 1972–2007 [37]. In West Kordofan, LULC changes were mapped for 2000–2005 [38]. Negative vegetation cover changes were reported in the two regions, mainly due to desertification and socio-economic effects. Similarly, in the Gedaref state, which is the focus of the present study, LULC conversion was evaluated by Sulieman [39] and Sulieman and Ahmed [40] in the southern (1972–2003) and Biro et al. [22] in the northern (1979–2009) regions, respectively. These studies found drastic changes in natural vegetation, mainly due to the areal extent of mechanized rainfed farming in Gedaref state. Furthermore, Sulieman [41] quantified LULC changes in the middle of Gedaref and found positive (in bare land) and negative (in dense forest cover) changes between the period 1973 and 2015. Notwithstanding, none of the above-mentioned studies have estimated the change in LULC in the entire Gedaref state, which is the country’s food basket. Moreover, no study has utilized a machine learning algorithm to classify LULC in rainfed agricultural areas in Sudan. Despite the relatively high LULC classification accuracy obtained in the previously-mentioned studies using ML classifier, the transferability of such a parametric mapping approach to other points in space and time could be hindered by overfitting due to limited training dataset; studies have yet to predict the future LULC changes in high productive rainfed agricultural areas in Sudan like Gedaref. Therefore, this paper aims to (1) assess LULC changes in the Gedaref state, Sudan for the past thirty years (1988–2018) using Landsat imageries and the RF classifier in Google Earth Engine (GEE) platform, (2) determine the underlying dynamics that cause the changes in the landscape structure using intensity analysis, and (3) predict future LULC in 2028 and 2048 using CA-ANN algorithm. This study is the first attempt to, at the same time, quantify LULC changes, estimate their intensities and predict the future LULC dynamics for the entire Gedaref state.

## Methodology

### Study area

**Fig 1:**
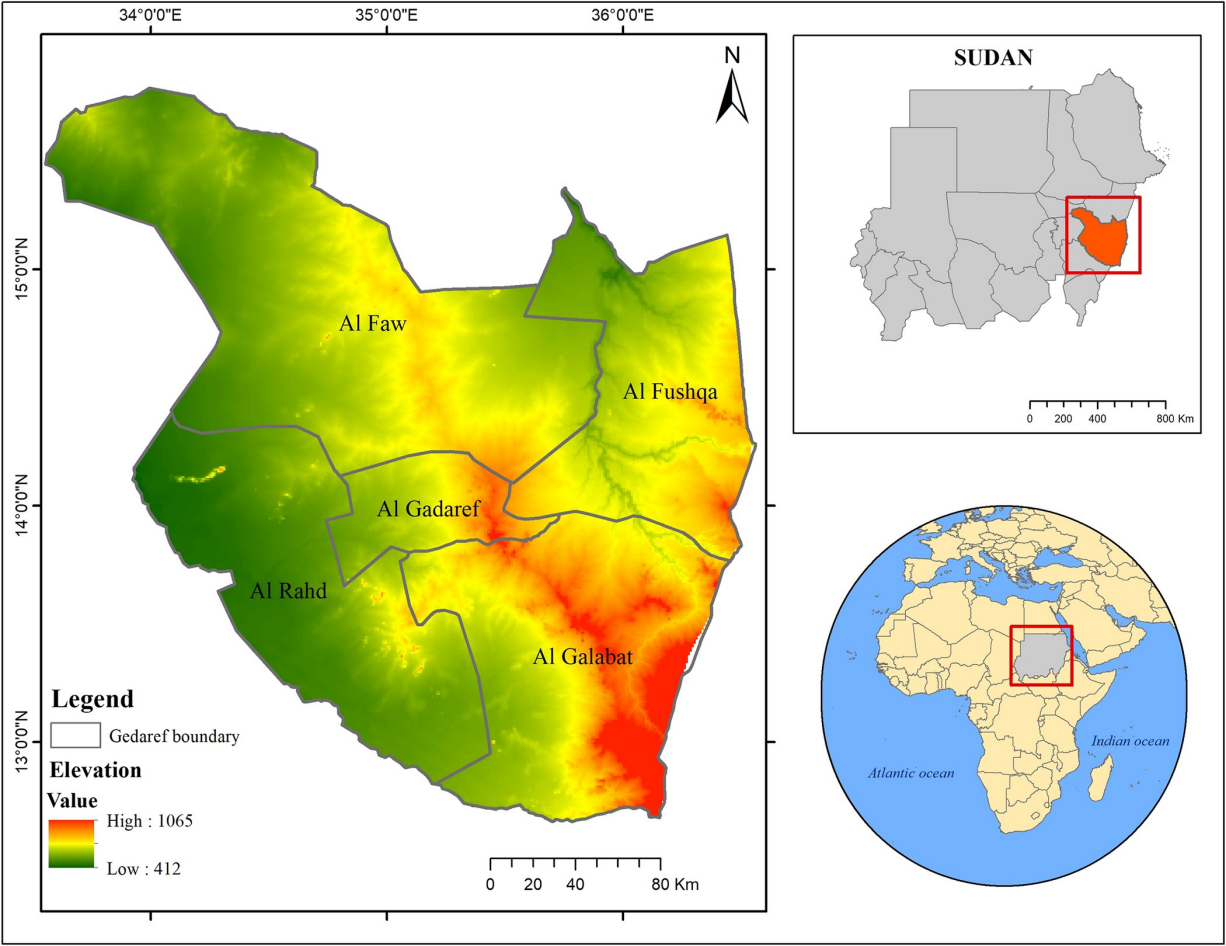
Location of Gedaref state in Sudan.

This study was conducted in Gedaref state, Sudan that expands between 33°– 37° E and 12°– 16° N (Fig 1). The study area covers approximately 78.228 km^2^ and is characterized by a semi-arid environment that receives an annual rainfall of 600 mm on average. The weather in Gedared state is relatively warm with a daily maximum temperature range of 25°C to 40°C [42]. The presence of fertile clay soils coupled with optimal amount of rainfall has made Gedaref state the most suitable region for crop cultivation under rainfed conditions in Sudan. Indeed, 80% of the population and households in the state mainly rely on different forms of agricultural activities for their livelihood [24, 38]. The major income generation activity in Gedaref state is crop cultivation, followed by animal keeping, and forest produce such as tapping of gum arabic and charcoal production [43]. The population in Gedaref state was estimated to be about 2,208,385 in 2018. Generally, in this state, the population grows by a rate of 4.7% annually, which is more than the national rate of 2.2% [44]. Thus, this region was chosen for this study due to its importance as Sudan’s hub of rainfed crop production, with moderately fertile soil and fairly good vegetation cover. However, the land in Gedaref has recently experienced dramatic degradation as the result of mechanized farming expansion, population growth and an extensive deforestation [22, 40].

### Data description

#### Satellite remote sensing data acquisition and pre-processing

**Fig 2:**
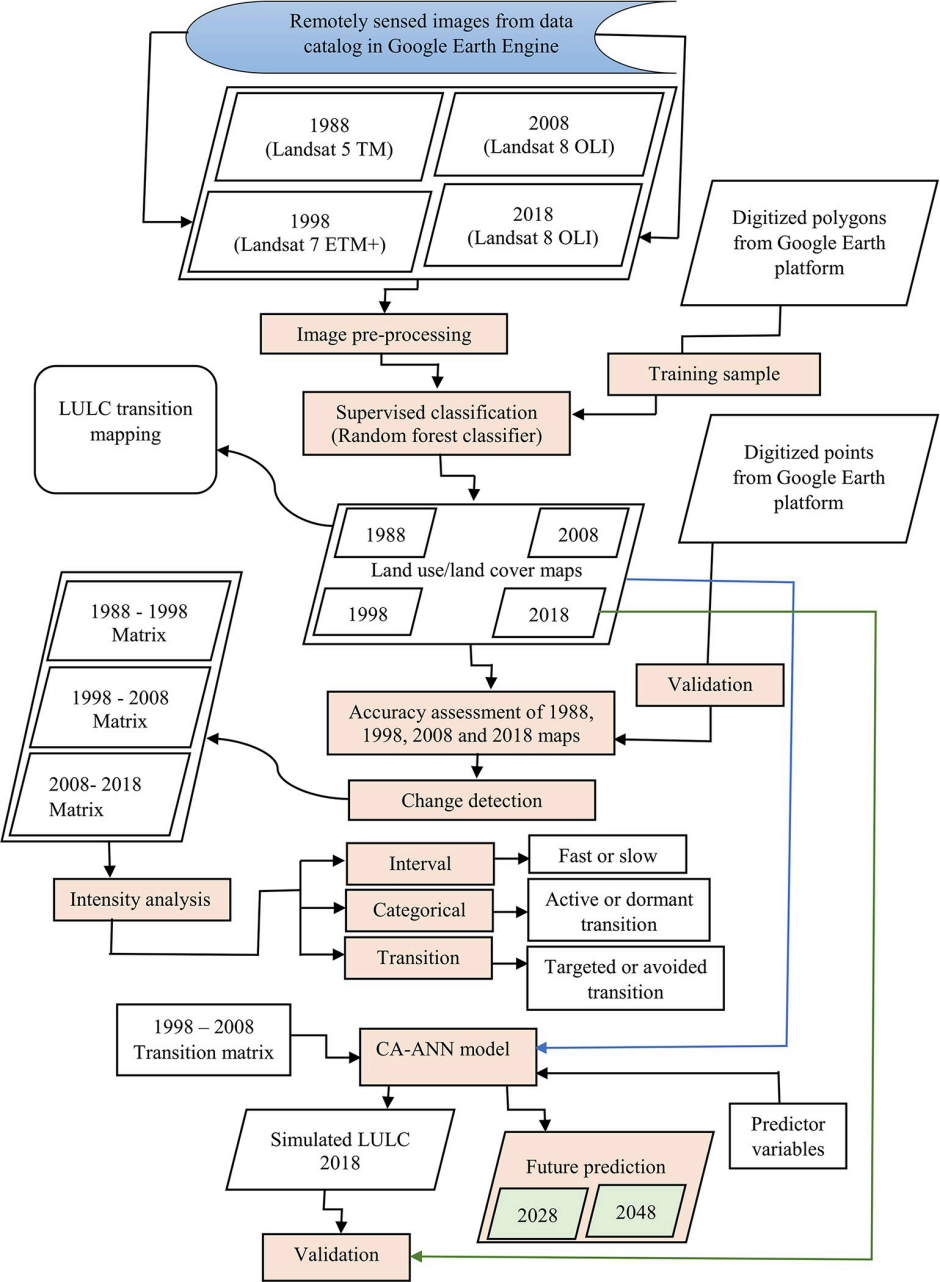
Methodological flowchart for the land use/ land cover (LULC) mapping, transition, intensity analyses and future prediction.

Fig 2 shows the methodological approach used in this study. Landsat multispectral images are the most widely used for time series analysis of LULC classification due to the long historical data that are available in their archive [45]. In this study, we used multi-date Landsat imagery for the years 1988, 1998, 2008 and 2018 acquired by Landsat 5 Thematic Mapper (TM), Landsat 7 Enhanced Thematic Mapper Plus (ETM+) and Landsat 8 Operational Land Imager (OLI) sensors from the freely available data catalog in GEE at a spatial resolution of 30 m in the World Geodetic System (WGS84) [46]. Standard image pre-processing, including cloud filtering, topographic, atmospheric, and geometric corrections, layer stacking and re-sizing was performed in GEE. A yearly (from 1^st^ January to 31^st^ December) median value was used to create a composite image for the selected years (i.e., 1988 and 1998 for Landsat 5, 2008 for Landsat 7, and 2018 for Landsat 8) [47, 48].

#### Training and testing data

The definition of LULC classes used in this study were based on the Intergovernmental Panel on Climate Change (IPCC) classes [49], namely cropland, forest land, grassland, water, and settlements. These five classes were obtained through onscreen digitization using historical high-resolution images on the Google Earth Pro platform. The onscreen digitization approach has been widely used and reported in the literature for obtaining LULC classes, and it is found reliable and accurate [50–52]. To obtain reasonably representative and reliable classification samples for each LULC class, we randomly collected 1000 on-screen reference samples for each year (1988, 1998, 2008 and 2018). From the 1000 reference samples, we created 700 polygons to train the classification algorithm (herein they are referred to as training data). These training polygons were relatively small in size, each contained many comparatively homogeneous pixels of a specific LULC class to reduce the influence of spatial autocorrelation. We used polygon samples as training data to capture the expected spectral intra and inter variability within and among the LULC classes. Moreover, polygon samples enable acquisition of the colour gradient within each LULC class (e.g., deep water against shallow water; high, medium, and low grassland coverage) to avoid confusion between the classes. From the remaining 1000 samples, we generated 300 data points, which were utilized to test the classification accuracy (herein they are referred to as test data). The test points were also randomly selected at a minimum distance of 100 m from the nearest training polygon to minimize overfitting and spatial multi-correlation [51].

### Landsat image classification

There are many advanced non-parametric machine-learning classification algorithms in GEE for supervised classification, such as RF, support vector machines, and classification and regression tree, among others [53]. Also, a meta-analysis of more than 300 peer-reviewed articles published in the last ten years before 2018 showed that the most used classifier for classifying satellite data in GEE is the RF [54]. The RF classification algorithm was employed in GEE to classify and distinguish among the LULC classes of Gedaref state in Sudan using the multi-date Landsat images. Many studies have reported that RF algorithm achieved higher classification accuracy and reliability compared to other machine learning algorithms [55–57]. This is because RF is a user-friendly algorithm that requires settings and optimization of two parameters only. It also can handle large and noisy data as well as outliers, and reduce overfitting. The algorithm can simulate missing values through the calculation of proximity among samples [58]. This algorithm is a combination of learning methods, which includes many individual decision trees [59, 60]. Each single decision tree (ntree) has many splits (mtry, i.e., number of randomly selected variables) and nodes that predict the final class label based on the large number of votes from all decision trees. Considering the recommendations of other studies [61, 62], we used 100 trees (ntree = 100), and a default mtry value (the square root of the number of predictor variables). The strength of RF is that it can efficiently process a huge number of input variables without being affected by outliers and noise in the data, and is highly robust against overfitting [51, 53, 63].

### Classification accuracy assessment

The reliability of a thematic LULC map relies on the overall and individual accuracies of the map and the individual classes, respectively [64]. Commonly, several metrics like kappa coefficient, producer’s accuracy (PA), user’s accuracy (UA) and overall accuracy (OA) are utilized to validate the accuracy of the thematic maps. In this study, we calculated these accuracy metrics, except kappa coefficient, to assess the accuracy of our LULC classification experiment. Subsequently, a class-wise accuracy metric was developed by applying the F1-score formula. This score combines PA and UA into a single fused accuracy measure ranging from 0 to 100% [65], and it was calculated using the equation below.


$${\left(F1\right)}_{i}=\frac{2\times {PA}_{i\;}\times {UA}_{i\;}}{{PA}_{i}+{UA}_{i}}$$ (1)

Additionally, due to many concerns regarding the use of kappa coefficient in evaluating the reliability of thematic maps [66], we performed two more suitable measures of disagreement *viz*., quantity disagreement (QD) and allocation disagreement (AD) that were proposed by Pontius and Millones [67]. The QD measures the difference between the observed and modelled class instances, whereas AD assesses the variance in the localities of the observed class samples.

### Land use/ land cover (LULC) change detection

LULC patterns of different time periods in the study area were assessed to detect the change in each class category. The change (%) for various LULC types in different point in time was calculated [68] as expressed in following equation:

$$\frac{C_{2}-C_{1}}{C_{1}}\times 100$$ (2)

where *C*_1_ and *C*_2_ are LULC class areas during the first (1988) and last of the study time period (2018), respectively.

The transition matrix produced in this analysis provided a general overview of the LULC stocks (amount and composition). Also, we evaluated the transfers among LULC categories every 10 years during the study period, 1988–1998, 1998–2008 and 2008–2018.

### Land use/ land cover (LULC) transitions mapping

In order to visually and quantitatively examine the nature of LULC transitions in Gedaref state and the transformation of each LULC class [13, 69], we used the Semi-Automatic Classification plugin that embedded in QGIS software version 2.18.15. The thematic LULC maps between 1988 and 2018 were used to create LULC transitions maps and their corresponding transition matrixes, where we used the LULC maps for the years 1988, 1998, and 2008 as reference layers to detect the transitions in each class in 30, 20 and 10 years (i.e., till 2018), respectively.

### Intensity analysis in land use/ land cover (LULC) transitions

Overall, the thematic LULC maps do not mimic the pattern and magnitude of the change that cause the landscape transformation [70]. To address this, Aldwaik and Pontius [71] proposed the so-called ‘intensity analysis’, which is a qualitative approach for better understanding the magnitude of the transformation in landscape structure. We performed LULC intensity analysis using the contingency table for each period to look at the extent and intensity of change at various scales; interval, category, and transition. The analysis of the interval level computes the rate and size of change over a specific point in time. Whereas, the categorical level analysis examines differences in the intensity of change across LULC classes. Lastly, the analysis of the transition level emphasises on the magnitude and direction of the change between the LULC categories in each time interval [70, 72].

The uniform intensity lines provided by all three levels of analysis depict a theoretical situation in which uniform transformation takes place across all LULC classes. The predicted class area from the interval level experiment defines the period that has annual fast or slow changes compared to the uniform intensity line. When the intensity of a category exceeds the uniform line, it is called an active category; when it falls lower than the uniform line, it is called a dormant category. Similarly, in the transition intensity, a targeted class is the one that its loss or gain exceeds the uniform intensity line. On the other hand, if a category does not reach the uniform intensity line, it is regarded as avoided [70]. Initially, we generated transition matrices of the periods 1988–1998, 1998–2008, and 2008–2018 for the thematic LULC maps. Thereafter, we used a tool developed by Aldwaik and Pontius [71] to compute the three intensity levels at different time intervals using the following equations and their description provided in Table 1:

**Table 1:** Mathematical symbols used to calculate different intensities as illustrated in Eqs 3–7 as described by Aldwaik and Pontius (2012).

Symbol	Description
*T*	number of time points
*γ*_*t*_	year at time point *t*
*t*	index for the initial time point of an interval [*γ*_*t*_−*γ*_*t*+1_],where *t* ranges from 1 to *T*−1
*J*	number of categories
*i*	index for a category at the initial time point of an interval
*j*	index for a category at the latter time point of an interval
*n*	index of the gaining category for the selected transition
*C*_*tij*_	size of transition from category *i* to category *j* during interval [*γ*_*t*_−*γ*_*t*+1_]
*S*_*t*_	annual change during interval [*γ*_*t*_−*γ*_*t*+1_]
*G*_*tj*_	intensity of annual gain of category *j* during interval [*γ*_*t*_−*γ*_*t*+1_] relative to size of category *j* at time *t*+1
*L*_*ti*_	intensity of annual loss of category i during interval [*γ*_*t*_−*γ*_*t*+1_] relative to size of category *i* at time *t*
*R*_*tin*_	intensity of annual transition from category *i* to category *n* during interval [*γ*_*t*_−*γ*_*t*+1_] relative to size of category *i* at time *t*
*W*_*tn*_	uniform intensity of annual transition from all non-*n* categories to category *n* during interval [*γ*_*t*_−*γ*_*t*+1_] relative to size of all non-*n* categories at time *t*

Firstly, the transitions at the interval level was computed (Eq 3), by dividing the magnitude of change by the length of time interval, generating percentage of spatial extent. The categorical annually gross loss intensity in a time interval was calculated, by dividing the size of the category’s annual gross loss by the size of the category at the beginning of each interval (Eq 4). On the other hand, the category’s annually gross gain intensity in a time interval was calculated by dividing the size of the category’s annually gross gain with the size of the category at the final stage of each time interval (Eq 5). The common hypothesis for each interval’s category level proposes that all categories experience gross loss and gross gain with the same annually intensity. This sum is equal to the transition rate in the interval (*S*_*t*_). If *L*_*ti*_ < *S*_*t*_, the loss of *i*, is paused during the interval *t*. In contrast, if *G*_*tj*_< *S*_*t*_, the gain of *j* is withheld during the interval *t*. In the case *L*_*ti*_ > *S*_*t*_, loss of *i* is considered to be active during the interval *t*; similarly, if *G*_*tj*_> *S*_*t*_, gain of *j* is considered active during that time interval. Eq (6) computes the annual transition intensity of the gain in a specific category *n* from other categories *i*, that is the amount of the annually transition to the specific category *n* from the other category divided by amount of another category at the beginning of each interval. The hypothesis at the level of transition for intervals states that particular category *n* moves to all other categories with a comparable annual intensity. This amount is calculated by dividing the size of the yearly gain of category *n* by the total quantities of sizes of all other categories at the beginning time of intervals (Eq 7). Hence, if *R*_*tin*_ < *W*_*tn*_, the gain of *n* pause *i* during the interval *t*. If *R*_*tin*_ > *W*_*tn*_, the gain of *n* targets *i* within interval *t*.


$$S_{t}=\frac{Change\;during\;{[\gamma }_{t},\gamma_{t+1}]}{\left(Duration\;of\;{[\gamma }_{t},\gamma_{t+1}])(Extent\;Size\right)}100\%=\frac{\sum_{j=1}^{J}[(\sum_{i=1}^{J}C_{tij})-C_{tij}}{(\gamma_{t+1},\gamma_{t})(\sum_{j=1}^{J}\sum_{j=1}^{J}C_{tij})}100\%$$ (3)

$$L_{ti}=\frac{Annual\;loss\;of\;i\;during\;{[\gamma }_{t},\gamma_{t+1}]}{Size\;of\;i\;at{\;\gamma }_{t}}100\%=\frac{[\sum_{i=1}^{J}C_{tij})-C_{tij}]/(\gamma_{t+1}-\gamma_{t})}{\sum_{j=1}^{J}C_{tij}}100\%$$ (4)

$$G_{tj}=\frac{\;Annual\;gain\;of\;j\;during\;[\gamma_{t},\gamma_{t+1}]}{\;Size\;of\;j\;at\;\gamma_{t+1}}100\%=\frac{\left[(\sum_{i=1}^{J}C_{tij})-C_{tij}]/(\gamma_{t+1}-\gamma_{t}\right)}{\sum_{i=1}^{J}C_{tij}}100\%$$ (5)

$$R_{tin}=\frac{Annual\;transition\;from\;i\;to\;n\;during\;{[\gamma }_{t},\gamma_{t+1}]}{Size\;of\;i\;at\;\gamma_{t}}100\%=\frac{C_{tin/}{(\gamma }_{t+1}-\gamma_{t})}{\sum_{i=1}^{J}C_{tij}}100\%$$ (6)

$$W_{tn}=\frac{Annual\;gain\;of\;n\;during\;{[\gamma }_{t},\gamma_{t+1}]}{Size\;of\;non-n\;at\;\gamma_{t}}100\%=\frac{[(\sum_{i=1}^{J}C_{tin})-C_{tnn})/(\gamma_{t+1}-\gamma_{t})}{\sum_{j=1}^{J}[(\sum_{i=1}^{J}C_{tij})-C_{tnj}}100\%$$ (7)

### Future land use/ land cover (LULC) prediction and validation

After generating LULC maps from Landsat data for the period 1988 to 2018 with 10 years intervals, future simulation of LULC change was performed using CA-ANN algorithm in MOLUSCE plugin that embedded in QGIS software version 2.18.15. Studies have shown that the CA-ANN model is more powerful and robust in simulating future LULC as compared to other models like linear regression and Marcov [31, 73, 74]. Moreover, the MOLUSCE plugin effectively processes LULC change analyses and is suitable for evaluating spatio-temporal LULC changes and predicting future scenarios [75, 76]. For future LULC predictions, we retained the same resolution (30 x 30 m) and WGS 84 coordinate system.

**Fig 3:**
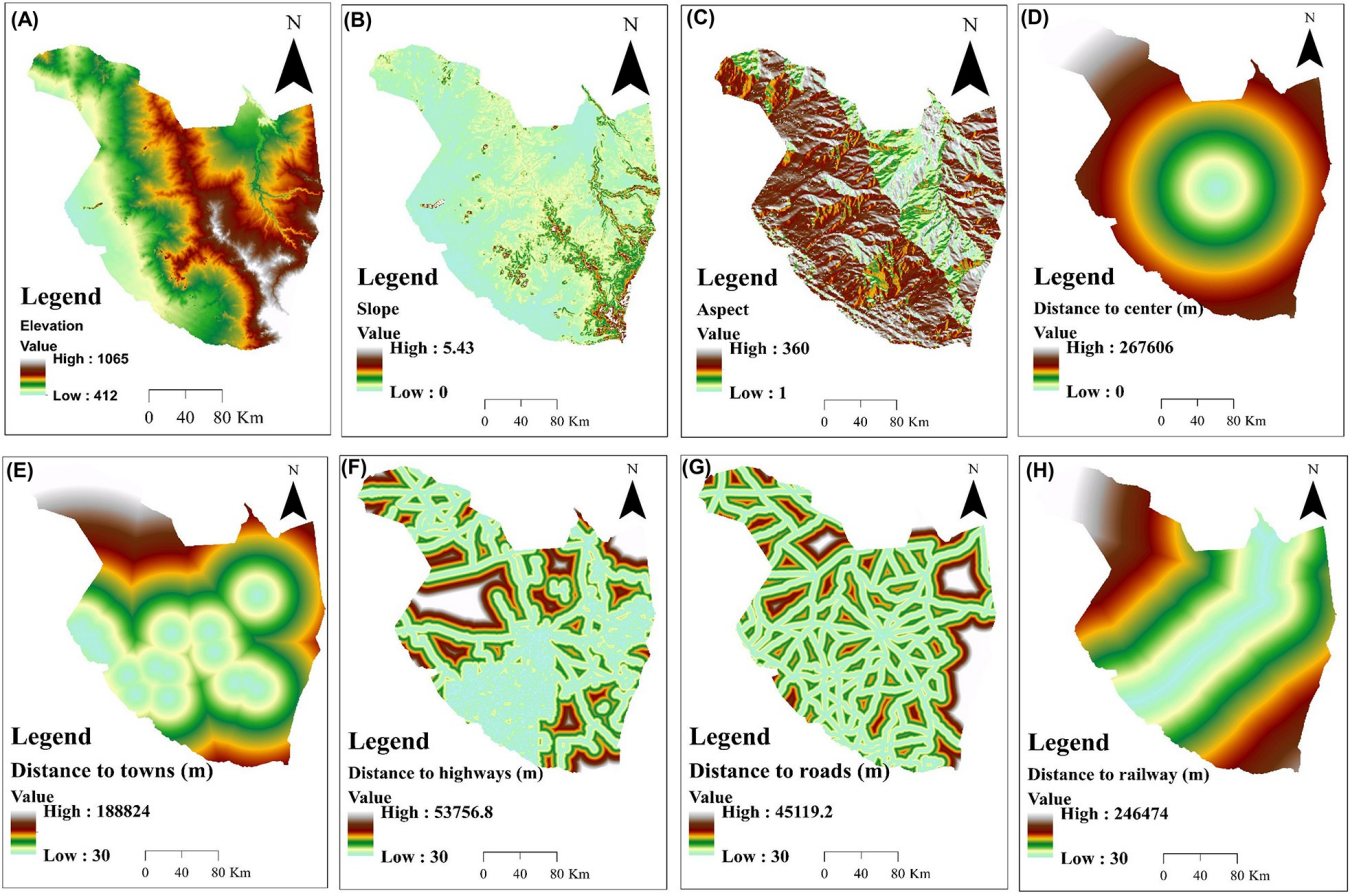
Predictor variables used for land use/ land cover (LULC) future prediction.

To simulate future LULC, it is recommended that a number of predictor variables, which play a major role in LULC change and transition should be considered [77]. Based on LULC change drivers that were reported in previous studies [78–80], and the availability of such factor datasets, we selected 8 predictor variables (Fig 3) to describe the LULC change processes that occurred in Gadaref state between 1988–2018. These predictors include topographic variables such as slope, aspect and elevation; and human disturbance variables like distance from Gedared state center, towns, highways, roads and the railway line (Fig 3). These variables are frequently used to predict LULC because they provide reproducible data on the natural and human disturbances in LULC processes [76].

Prediction of future potential LULC for a prospective project can be only reliable if the simulation outcome is validated using existing datasets. Accordingly, in the first step, we simulated LULC for the year 2018 using the transition matrix generated from the thematic maps of the years 1998 and 2008 and the selected predictor variables that are presented in Fig 3. Thereafter, the validation process was performed using a comparative analytical procedure of the overall correctness percentage and kappa coefficient in the MOLUSCE plugin. Specifically, to validate the performance of CA-ANN model, we compared the simulated LULC map for 2018 that was generated using CA-ANN algorithm with the one that generated for the same year using the multi-date Landsat images and RF classifier.

After obtaining adequate validation metrics, we utilized LULC data from 2008 and 2018 maps (herein referred to as prediction data) to simulate future LULC in 2028 and 2048.

## Results

### Land use/ land cover (LULC) classification

**Fig 4:**
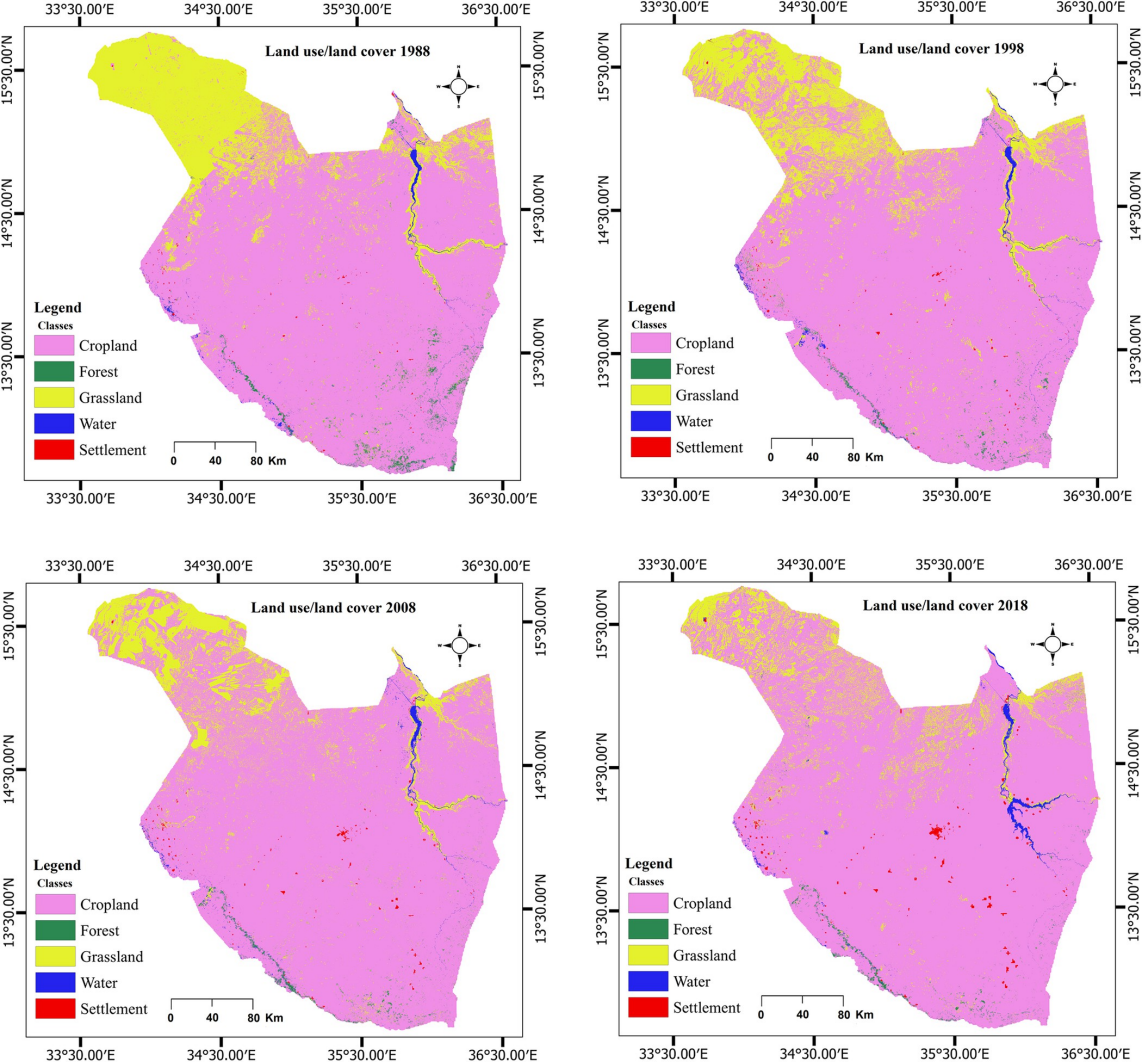
Classified land use/ land cover (LULC) maps of Gedaref state for the years 1988, 1998, 2008 and 2018 produced using multi-date Landsat images and random forest classification algorithm.

The classified LULC maps and associated area statistics under each class category for 1988, 1998, 2008 and 2018 are presented in Fig 4 and Table 2. Among the LULC categories, cropland was the most dominant in 1988, followed by grassland, each occupying 78.64%, and 19.64%, respectively of the total area (Table 2). Whereas, forest, water and settlement covered less than 2% of the studied landscape (Table 2). A similar trend was observed for the other studied years. Nevertheless, there were distinct LULC change in Gedaref during the study period where an expansion in cropland and settlement area, and a decline in forest and grassland areas were observed (Fig 4 and Table 2). Water area increased in all study years, except in 2008.

**Table 2:** Area (ha) and percent cover (%) of each land use/ cover (LULC) class in Gedaref state for the years 1988, 1998, 2008, and 2018 estimated using multi-date Landsat images and random forest classification algorithm.

Year	1988		1998		2008		2018	
	Area		Area		Area		Area	
**LULC**	ha	%	ha	%	ha	%	ha	%
**Cropland**	5023958.04	78.64	5289566.13	82.8	5566904.46	87.14	5723662.45	89.59
**Forest**	0069053.13	01.08	0037217.16	00.58	0035406.99	00.55	0030056.44	00.47
**Grassland**	1254479.85	19.64	1012649.94	15.85	0734874.57	11.51	0548998.87	08.59
**Water**	0034132.05	00.53	0039363.12	00.62	0035961.84	00.56	0057366.22	00.89
**Settlement**	0006739.02	00.11	0009565.74	00.15	0015214.23	00.24	0028278.13	00.44
**Total**	**6388362.09**	**100**	**6388362.09**	**100**	**6388362.09**	**100**	**6388362.09**	**100**

### Accuracy of land use/ land cover (LULC) classification

The accuracy evaluation metrics of the classified LULC maps generated from confusion matrix is presented in Table 3. The overall accuracy of the 1988, 1998, 2008 and 2018 LULC maps was 81.75%, 83.28%, 85.15%, and 87.70%, respectively. While F1 score value for all LULC classes in all years ranged between 80% and 90% (Table 3). Additionally, the results of QD for all the classified maps ranged between 3% and 4%, whereas, AD varied from 9% to14% (Table 3).

**Table 3:** Overall and individual class accuracies of land use/ land cover (LULC) maps of Gedaref state for the years 1988, 1998, 2008 and 2018.

Class	Producer’s accuracy (%)	User’s accuracy (%)	F1 score (%)	Overall accuracy (%)	Allocation disagreement (%)	Quantity disagreement (%)
**1988**						
**Cropland**	88.88	77.41	82.75	81.75	14	4
**Forest**	81.13	81.39	81.39			
**Grassland**	85.29	80.55	82.85			
**Water**	81.13	81.13	81.13			
**Settlement**	73.13	89.09	80.32			
**1998**						
**Cropland**	83.07	83.07	83.07	83.28	14	3
**Forest**	83.13	87.34	85.18			
**Grassland**	84.48	77.77	80.99			
**Water**	86.66	81.25	83.87			
**Settlement**	78.78	86.66	82.53			
**2008**						
**Cropland**	95.23	82.19	88.23	85.15	11	4
**Forest**	86.07	85.00	85.53			
**Grassland**	76.56	87.50	81.63			
**Water**	88.88	87.67	88.27			
**Settlement**	76.92	83.33	80.00			
**2018**						
**Cropland**	89.28	80.64	84.74	87.70	9	3
**Forest**	89.07	88.33	88.70			
**Grassland**	84.78	81.25	82.97			
**Water**	79.20	94.11	86.02			
**Settlement**	92.40	88.48	90.40			

### Land use/ land cover (LULC) change detection

Forest and grassland categories were considerably decreased by about 46% and 19%, respectively in the first 1998–2008 interval (Table 4). On the other hand, there was an expansion in cropland area during the whole study period, which ranged between about 2.8% and 5% in the three intervals. Similarly, the settlement areas dramatically increased by 41.94% and 59.04% in the first and second intervals, respectively, with the third interval being drastically greater (85.86%) than that of the first and second intervals. Water class increased by 15.32% in the first period of the study and decreased during the second time period, with a sharp increase (85.86%) in the third interval compared to the increase in the first interval.

**Table 4:** Land use/ land cover (LULC) change estimates (area and percentage) for Gedaref state for the 1988–1998, 1998–2008, 2008–2018 and 1988–2018.

Year	1988–1998		1998–2008		2008–2018		1988–2018	
**LULC**	Area		Area		Area		Area	
	ha	%	ha	%	ha	%	ha	%
**Cropland**	265608.00	5.28	277338.30	05.24	156758	2.81	699704.35	13.92
**Forest**	-31835.97	-46.10	-1810.17	-04.86	-5350.55	-15.11	-38996.70	-56.47
**Grassland**	-241829.91	-19.27	-277775.37	-27.43	-185875.71	-25.29	-705480.98	-56.23
**Water**	5231.07	15.32	-3401.28	-8.64	21404.38	59.52	23234.17	86.07
**Settlement**	2826.72	41.94	5648.49	59.04	13063.9	85.86	21539.11	319.61

### Land use/ land cover (LULC) transitions mapping

**Fig 5:**
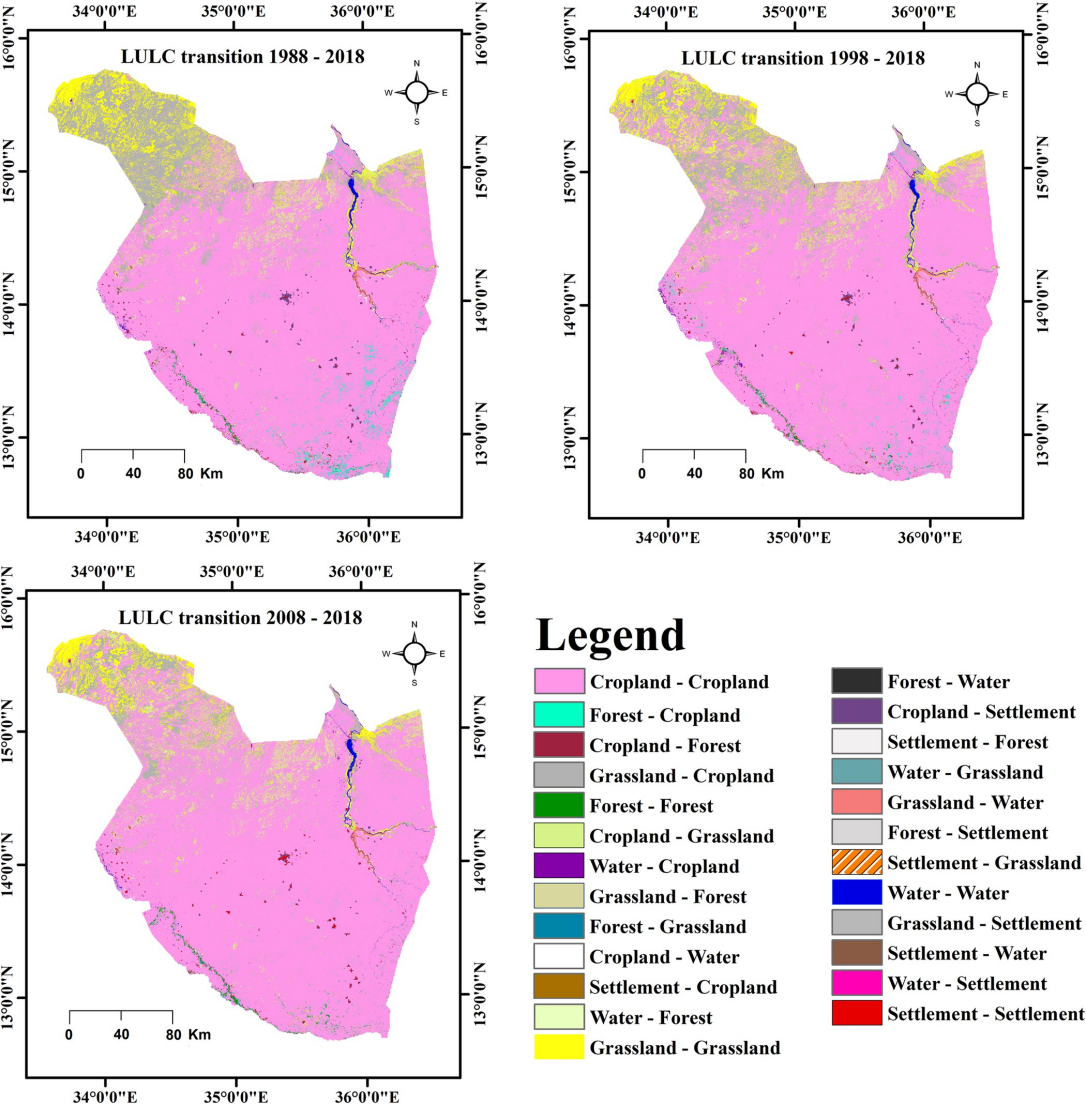
Land use/ land cover (LULC) transitions of Gedaref state during 1988–2018, 1998–2018 and 2008–2018.

The results in Fig 5 and S1–S3 Tables indicate the transformation of each of the five LULC classes during 1988–2018, 1998–2018 and 2008–2018. The major LULC transition that took place over the study period (1988–2018) were forest to cropland, grassland to cropland, cropland to grassland, water to cropland, cropland to water, cropland to settlement and grassland. In particular, the dominant transition in Sothern and Western parts of Gedaref sate across the three transition periods (1988–2018, 1998–2018 and 2008–2018) was forest to cropland. Whereas the transition from grassland to cropland was mainly observed in Northern and Northeast of Gedaref state (Fig 5). The transition from cropland to settlement was mainly occurred in Central, Northwest, and Southern parts of Gedaref state.

### Intensity analysis in land use/ land cover (LULC) transitions

**Fig 6:**
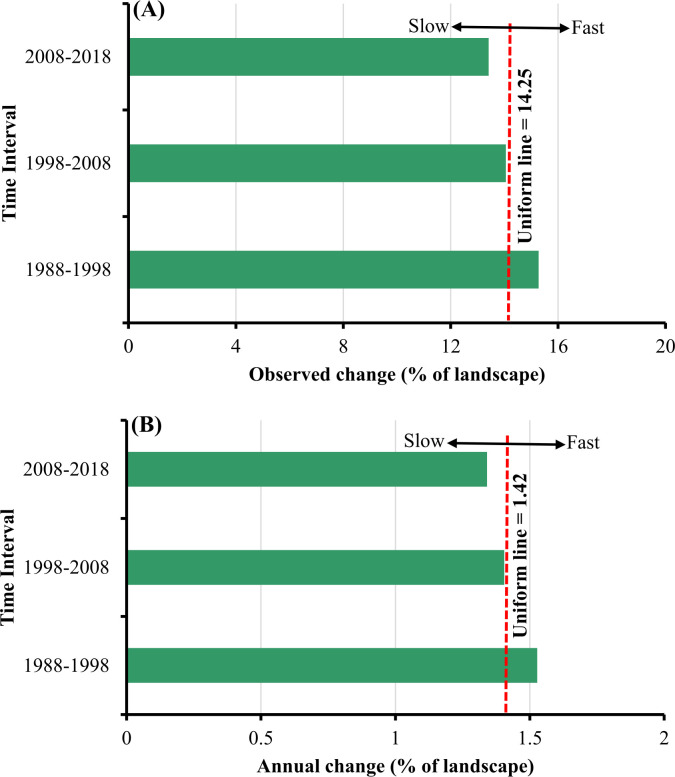
Interval level intensity of land use/ land cover (LULC) change for 1988 1998, 1998–2008 and 2008–2018. A) the percent of the area that changed over each interval and B) the percentage of the area that annually changed during each interval.

Interval level intensity results showed the changing intensity over each time period (Fig 6A) and the annual change between intervals (Fig 6B). Interval analysis revealed that the period 1988–1998 experienced fast LULC transitions and annual change rate. In the second interval, the observed and annual transitions were relatively equal to the uniform line but lower than in the third interval indicating slow LULC transitions.

**Fig 7:**
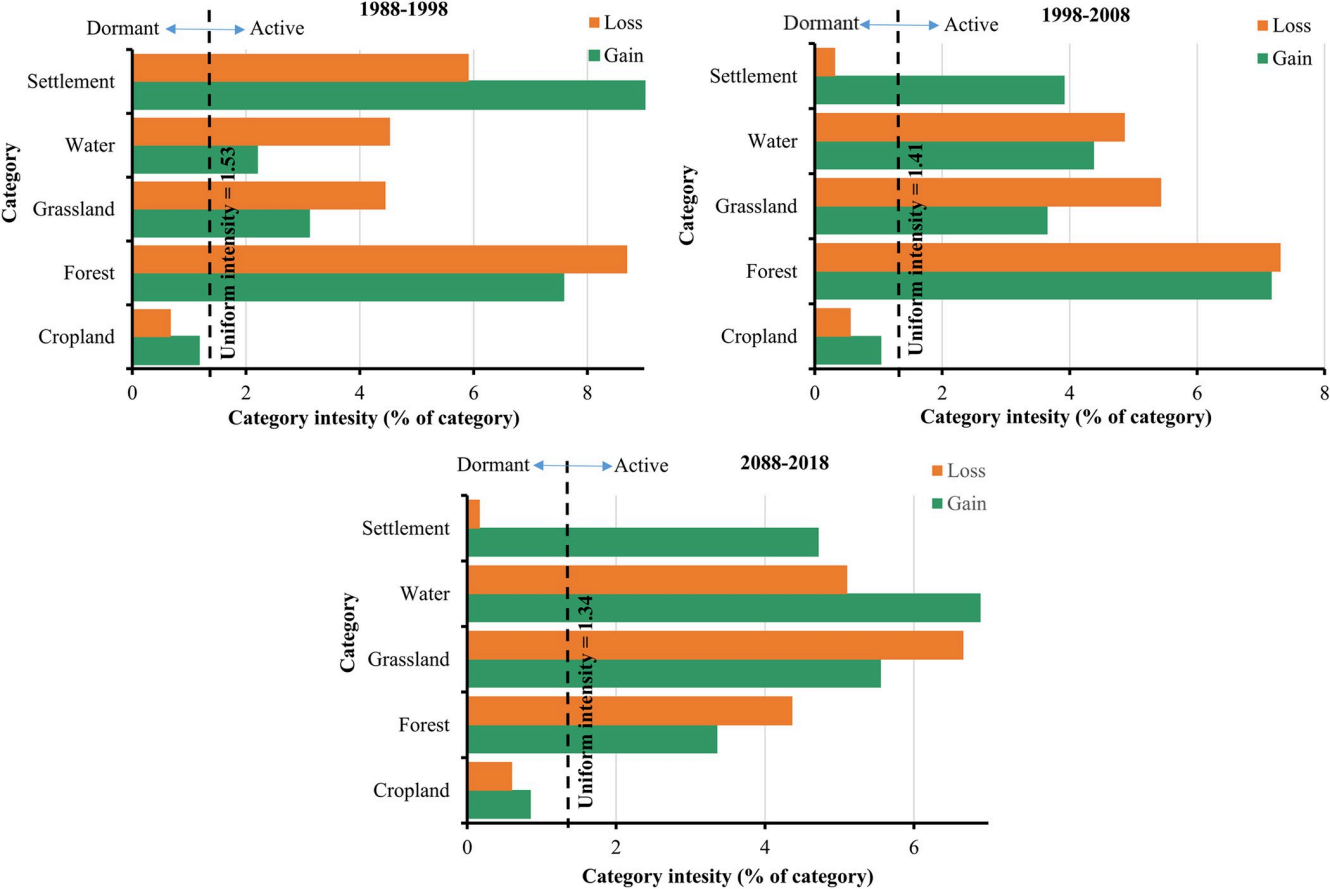
Land use/ land cover (LULC) category level intensity for 1988–1998, 1998–2008, 2008 and 2018.

Fig 7 illustrates that the LULC classes experienced dormant or active changes during the study period. Moreover, it shows that the active LULC classes were the ones that their gain or loss is crossing the uniform line. In contrast, the dormant categories are those of gain or loss that do not reach the uniform line. During the three intervals, forest, grassland, water and settlement categories were active gainers with relatively higher gains in the settlement, forest and water, respectively. However, cropland category was the dormant gainer throughout the study period. Three categories, i.e., forest, grassland and water were active losers during the three intervals with relatively higher losses in forest and grassland, respectively. Whereas, settlement was an active loser during the first interval and a dormant loser during the second and third intervals. The cropland category was the dormant loser during the three intervals.

**Fig 8:**
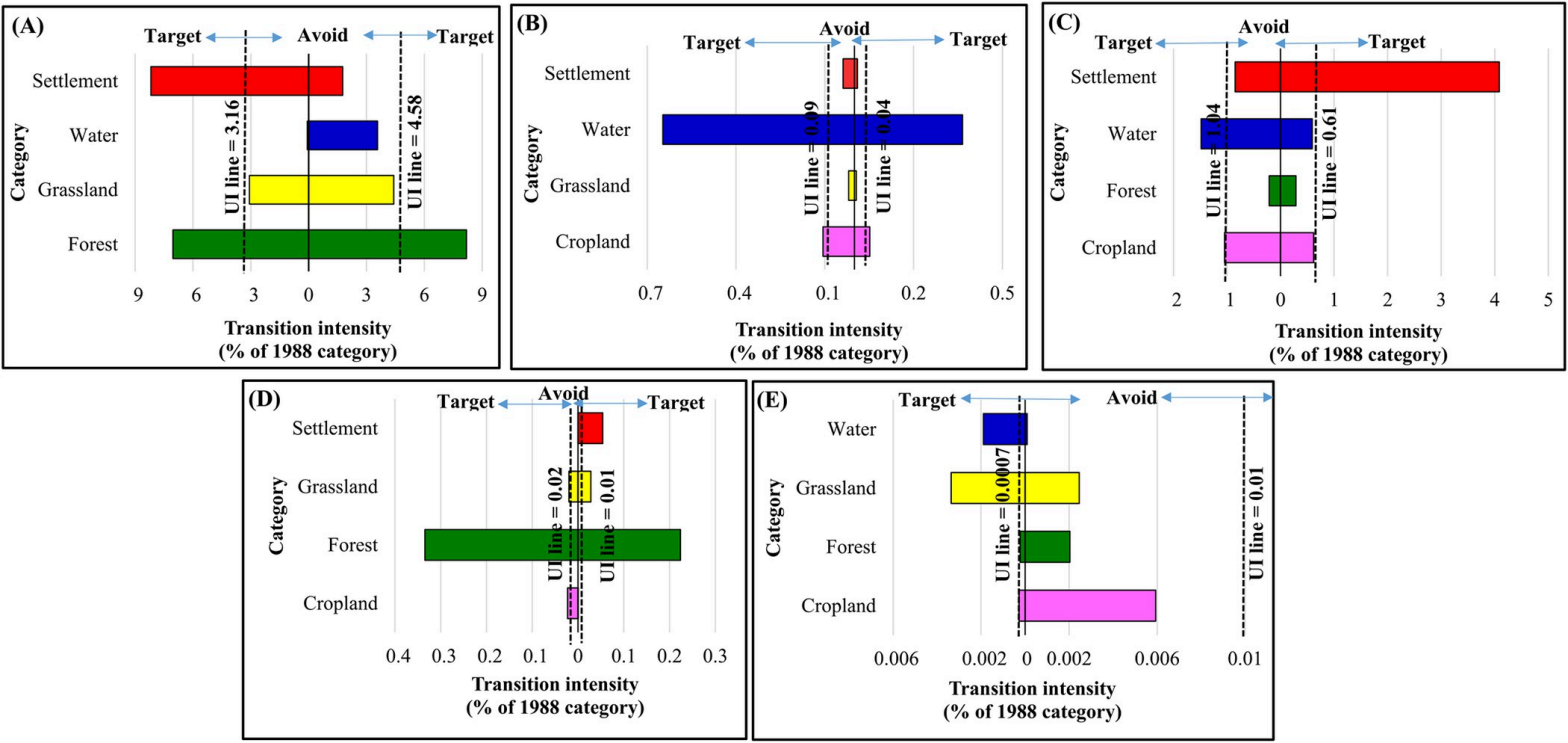
Transition level intensity of land use/ land cover (LULC) for the period 1988–1998. (A) cropland, (B) forest, (C) grassland, (D) water and (E) settlement (gains on the right and losses on the left).

**Fig 9:**
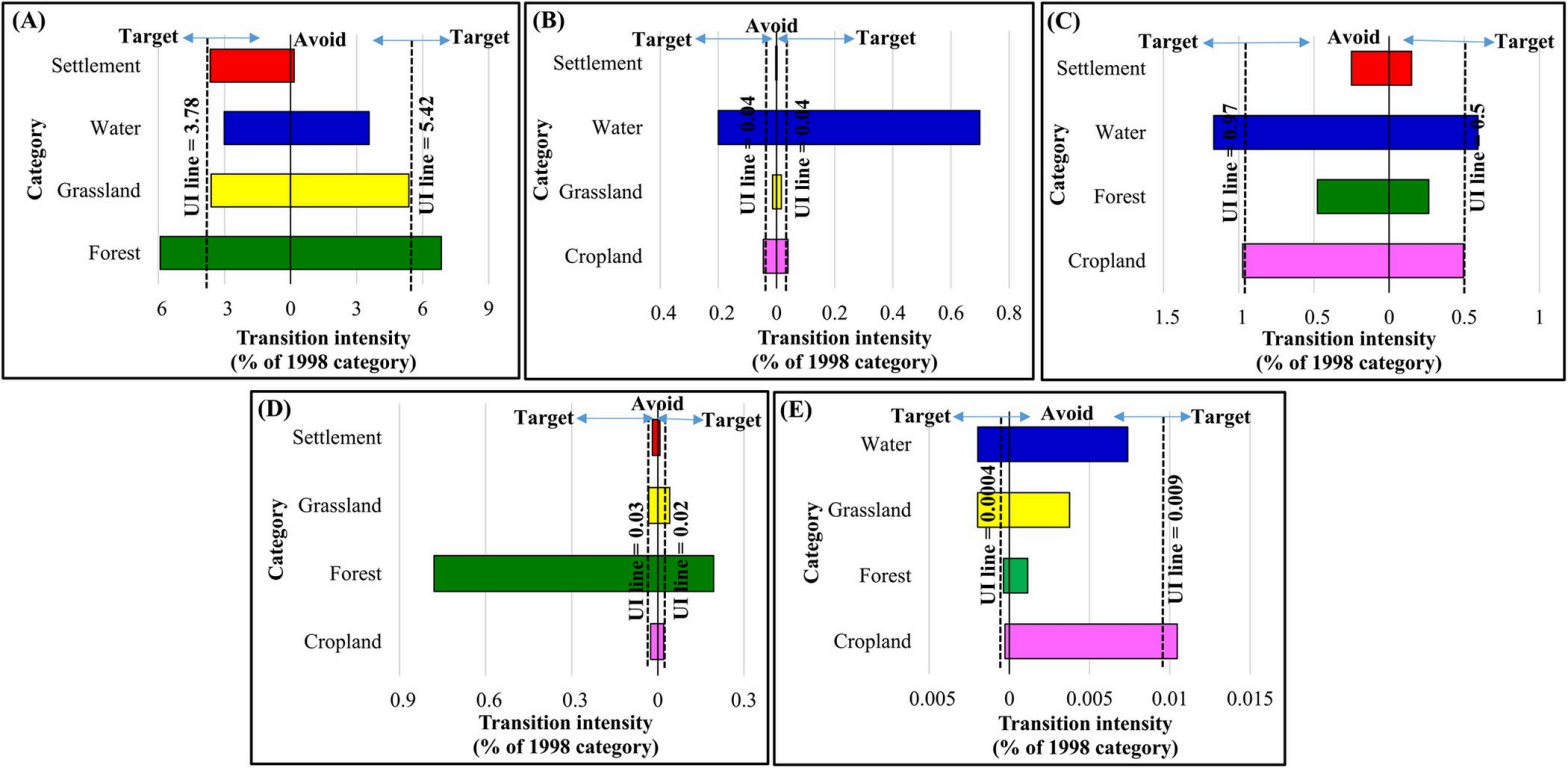
Transition level intensity of land use/ land cover (LULC) for the period 1998–2008. (A) cropland, (B) forest, (C) grassland, (D) water and (E) settlement (gains on the right and losses on the left.

**Fig 10:**
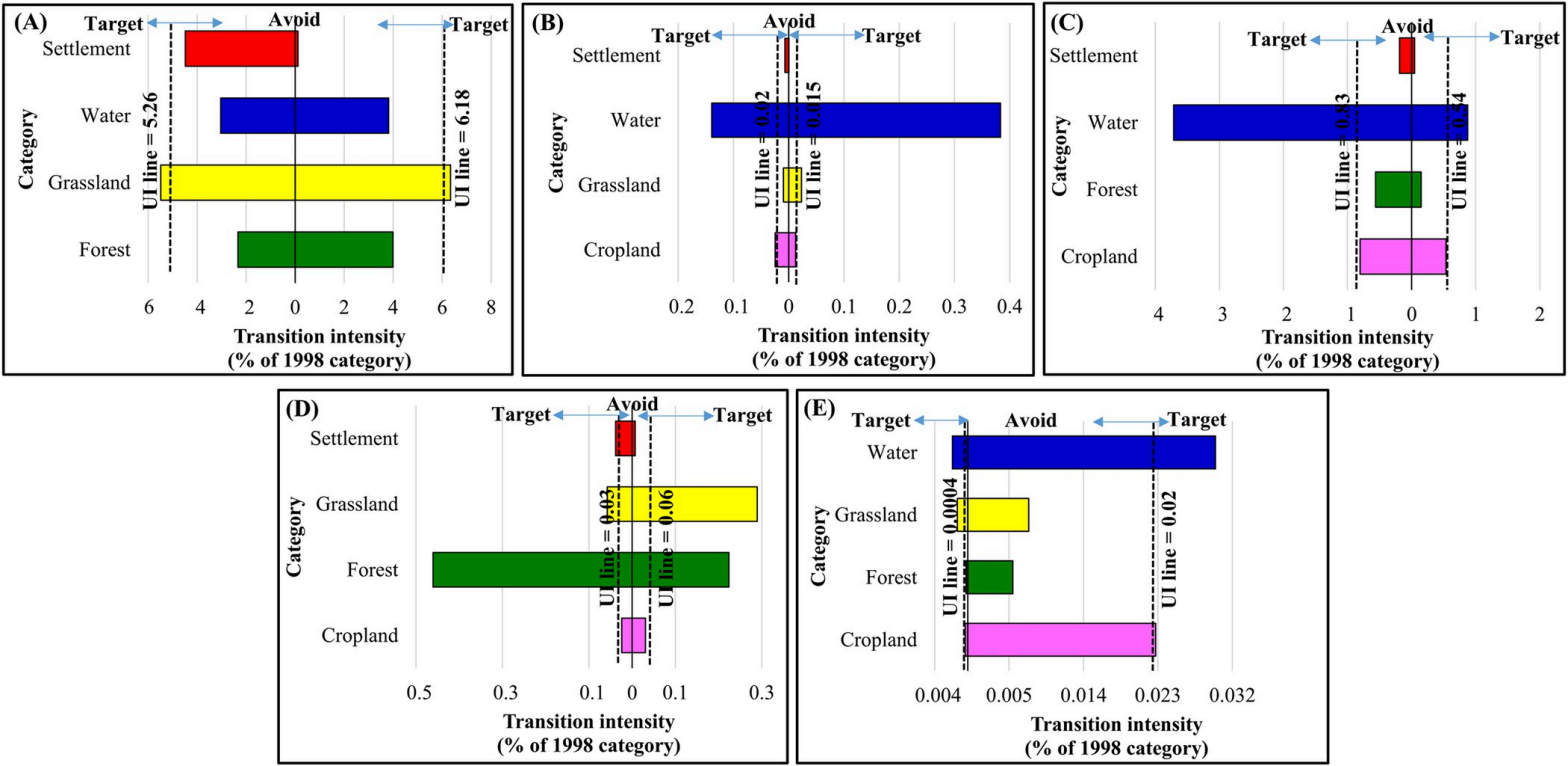
Transition level intensity of land use/ land cover (LULC) for the period 2008–2018. (A) cropland, (B) forest, (C) grassland, (D) water, and (E) settlement (gains on the right and losses on the left).

Figs 8–10 demonstrate the results of the transition level analysis for each of LULC category. The vertical dashed lines on both sides of the chart represent hypothetical uniform intensity lines. The left side of the uniform line explains the theoretical uniform value in the transition intensity that accounted for the losses in specific LULC class. While the side on the right represents the gains in transition intensity. The intensity showed that the expansions in cropland in 1998 targeted forest only and losses in cropland targeted both forest and settlement (Fig 8A). The gain and loss in forest areas targeted water and cropland (Fig 8B). Likewise, the transition to grassland in 1998 targeted settlement and avoided the other LULC categories, while the losses in grassland targeted water (Fig 8C). Losses in water targeted cropland and forest, whereas the gains in the water category targeted forest, grassland, and settlement (Fig 8D). The reductions and expansions in cropland between 1998 and 2008 followed a similar trend to that of 1988–1998, where this category targeted forest areas (Fig 9A). On the other hand, the expansions in settlements in the same period targeted cropland, but reductions in settlement area targeted grassland and water equally (Fig 9E). Gains in water targeted forest and slight grassland, and losses targeted forest (Fig 9D).

In the final ten years of the studied period (2008–2018), cropland had different transition intensity trends from the first and second periods and relatively similar transition intensity trends for the forest. In contrast, during this period, water experienced an expansion with transition intensity targeting forest and grassland (Fig 10). The intensity of water gained from grassland was more significant than for forest. Settlements expansion over this period targeted water and marginally cropland (Fig 10). Losses in settlement targeted water and grassland with marginal avoidance in cropland. In both cases of losing and gaining in the settlement, the highest transition intensity was from water and to water (Fig 10).

### Future prediction of land use/ land cover (LULC)

**Fig 11:**
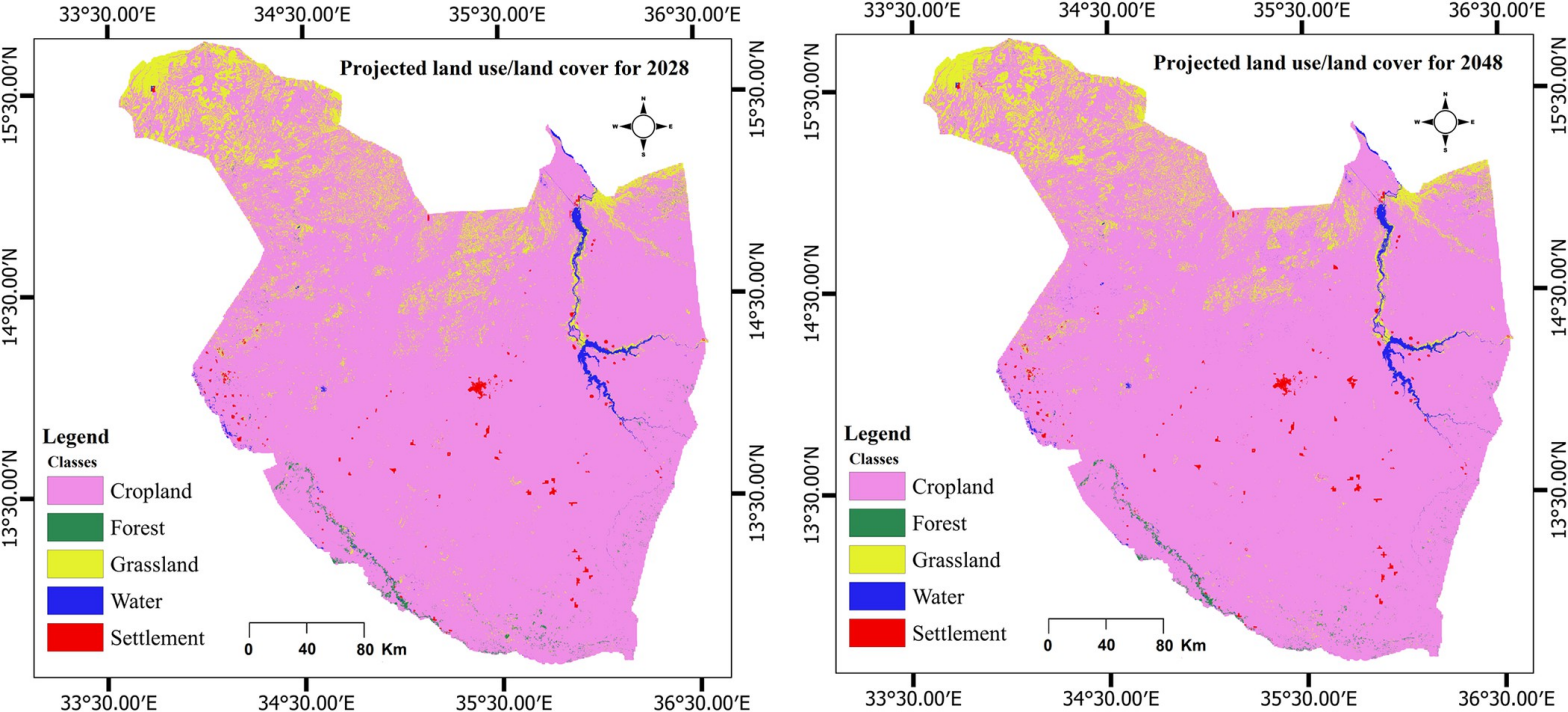
Predicted land use/ land cover (LULC) in Gadaref state for 2028 and 2048.

The CA-ANN predicated future LULC for Gedaref state for the years 2028 and 2048 (Fig 11) based on the LULC maps of the years 2008 and 2018 that were generated from Landsat images (Fig 4). The model predicted a slight increase in cropland area from 89.59% to 90.43% and a considerable decrease in forest area (0.47% to 0.41%) between 2018 and 2048 (Table 5). The model also predicted a marginal decrease in grassland (8.59% to 7.78%) and an increase in settlement area from 0.44% to 0.50%. Whereas, water area was predicted to be relatively consistent (0.89% to 0.88%) (Table 5) between 2018 and 2048.

**Table 5:** The proportion of predicted land use/ land cover (LULC) categories at Gedaref state.

Year	2018		2028		2048	
	Area		Area		Area	
**LULC**	**ha**	**%**	**ha**	**%**	**ha**	**%**
**Cropland**	5723662.45	89.59	5731266.73	89.71	5777105.53	90.43
**Forest**	0030056.44	0.47	0029882.92	0.46	0025895.38	0.41
**Grassland**	0548998.87	8.59	0541367.59	8.48	0496928.83	7.78
**Water**	0057366.22	0.89	0057093.79	0.89	0056335.45	0.88
**Settlement**	0028278.13	0.44	0028751.08	0.45	0032096.92	0.50
**Total**	**6388362.09**	**100**	**6388362.09**	**100**	**6388362.09**	**100**

The validation results of the CA-ANN algorithm showed that the model adequately simulated the future LULC pattern in Gedaref state with an overall correctness of 87% and kappa coefficient of 0.86. This indicate that the simulated LULC map of the year 2018 was highly comparable to the actual 2018 that produced in this study, indicating an accurate model performance.

## Discussion

In the present study, we combined on-screen digitization of polygons and points from Google Earth Pro platform, RF classification algorithm and dense multi-date Landsat satellite images (1988–2018) to map LULC types in Gedaref state, Sudan. For the first time, LULC for 1988–2018 was mapped in the entire Gedaref state, and its future for the years 2028 and 2048 predicted. In addition, this is the first attempt to evaluate LULC rate of change, intensity and transition in Gedaref for 30 years (1998–2018). Our methodology has several advantages as acquiring on-screen polygons and points from Google Earth can reliably be utilized to accurately characterize landscape structure, especially for satellite images that have a medium spatial resolution [70, 81, 82]. Also, for historical satellite image classification, real-time ground truthing observations might not be available, hence freely accessible platforms like Google Earth can provide accurate reference data for classification experiments. To minimize the expected spatial autocorrelation, we used point observations with a distance not less than 100 m from the nearest training polygon to validate the classified LULC maps. Hence, our mapping approach provided accurate LULC patterns for Gedaref state, which has a dynamic landscape setup.

The overall accuracies for the years 1988, 1998, 2008, and 2018 images ranged between 81.75% and 87.70%, which are greater than a recommended acceptable LULC classification accuracy of 70% [83, 84]. Nevertheless, the progressive increment in our LULC classification accuracy from the initial study year (1988) to the last year (2018) could indicate challenges associated with the availability of historical images of relatively better spatial resolution from Google Earth in the past decades.

The results showed that cropland was the most dominant LULC class covering between 78.64% and 89.59% of the total acreage of Gedaref state. This is due to the fact that Gedaref region is the main rainfed agricultural area of Sudan, where about 80% of the population in this state mainly depend on agricultural production for their livelihood [20]. In addition, clay soil, and the high amount of rainfall (400–800mm) that characterize Gedaref state, offer the optimum conditions for the cultivation of many food and cash crops such as sorghum, millet, sunflower, sesame, and cotton. This also explains the domination of this area by agricultural land. Our findings also revealed accelerated LULC change between 1988 and 2018, with an expansion in cropland and settlement, and a decrease in forest and grassland areas. These land use dynamics could be due to human activity such as the horizontal expansion of settlements and cultivated areas, not only by the local communities but, also by the investors and other communities from the neighbouring states and countries [85]. Although there was a substantial increase in cropland, the change in water class was unsystematic over the study period with a dramatic increase between 2008–2018 (59.52%). This could be explained by the fluctuation in the amount of rainfall that was observed in the study area due to climate variability and change [20].

Specifically, cropland has increased from 78.64% to 89.59% during the study period between 1988 and 2018, while the forest area declined from 1.08% to 0.47% of the total area in Gedaref state. Our findings agreed with the results of Gadallah et al. [42], who reported a decrease of forest area in Wad Albashir forest in Al Rahad locality, Gedaref state from 72.2% in 2001 to 58% in 2017, whereas cropland increased from 25.9% to 38.3% over the same period. This has been confirmed by our transition mapping, which showed a huge transformation of forested area to agricultural land, particularly along Al Rahad River where Wad Albashir forest is located, and in other forested areas in the southern Gedaref state. Generally, the decline in the forest area could be linked to agricultural expansion, firewood, charcoal production, timber, construction, flooding, soil erosion and desertification. The decline in grassland (-56.23%) from 1988 to 2018 in Gedaref region confirms the results of other studies that were conducted in some parts of the state [24, 86]. This decrease can also be explained by the fact that agricultural areas usually expanded at the expense of grassland. Also, the transition analysis showed substantial transformation of agricultural area at expense of grassland mainly in the northern and northern-eastern parts of Gedaref state. The regulations and policies of land use in Gedaref are biased toward cropland, compared to the grassland that was used by the pastoralist for grazing. A study by Sulieman and Elagib [24] reported that this bias was adopted in the solution of disputes during the British colonial period when the Soil Conservation Committee recommended in 1944 "where nomadic pastoralists were in direct competition for land with settled cultivators, the rights of the cultivator should be considered as paramount because his crops yield a higher return per unit area". Although new laws and regulations were put in place in Gedaref state, the 1944 recommendation still implemented in some areas. Our results also showed that the settlement areas drastically expanded more than three times (319.61%) between 1988 and 2018. This expansion was mainly took place in central Gedaref where the capital city is located and along Atbara, Al Rahad, Saiteet, and Basalam Revisers. Likewise, the southern and western parts of the state, where the major agricultural schemes are exist, experienced the same transformation from cropland to settlement. The expansion in settlement is primarily to meet the demand for shelter for the raid population growth and the increase of industrial areas in the region. This is coupled with the increase in cropland, which can be explained by the rising demand for food to meet the growing population. Biratu et al. [87] reported an increase in cropland and settlement areas in Ethiopia between 1986 and 2021 because of the growing population’s demand to ensure food and nutrition security in the country.

The unsystematic change in water class in our study could be explained by many seasonal waterways and rivers such as Atbara, Al Rahad, Saiteet, and Basalam flowing northward from the Ethiopian highlands in the rainy season, which differ from one year to another. However, the high increase in water class between 2008 and 2018 is associated with the construction of the Upper Atbara and Saiteet Dam complex, which is a twin dam consisting of two dams: Rumela Dam on the Upper Atbarah River and Burdana Dam on the Saiteet River [88]. Construction of the dam began in 2011 and completed in 2016, intending to provide irrigation water for agriculture, supply potable water for eastern states of Sudan, and power generation.

The interval level intensity revealed an intensive change of LULC in the first 10 years (1988–1998) compared to the second (1998–2008) and third (2008–2018) intervals. However, change in 2008–2018 was slower than in 1998–2008. This indicates that the impacts of socio-economic and physical driving factors during the three decades were different. This also implies a rapid change in Gedaref landscape in the first decade of our study period. The rapid LULC changes correspond to the areal extent of mechanized rainfed farming in the area since the late 1970s, which has attracted some migrants from different parts of the Sudan leading to rapid change in LULC over the first interval. A study by Miller [89], reported that the development of mechanized agricultural and grain trade in Gedaref during the 1970s enhanced the immigration to the state from different parts of Sudan, which increased socio-economic activities in the region.

The categorical intensity analysis; main findings firstly shown that forest, grassland and water were both active losers and gainers. This explains that gains and losses of these categories happen at intensities greater than the average intensity of all LULC categories. The intensity of active losses and gains in grassland and water could be explained by the associated seasonality of these two categories with the rainy season in Gedaref state. In contrast, the active gaining and losing in forest can be explained by the attempts of controlling the invasive Mesquite trees (*Prosopis juliflora*). This plant rapidly invades and colonizes the uncultivated land and farmers mostly cut down Mesquite trees to clear the land for cultivation, which might result in a fluctuation in forest cover. Secondly, the settlement was an active gainer and losier category between 1988 and 1998, and an active gainer and dormant loser in the 1998–2008 and 2008–2018 periods. This could be linked to lack of visual clarity of settlement pixels when reference data were gathered from Google Earth platform. In addition, the nature of the hut houses (locally called quttiyya), which are made of wood, grass and reeds might have been mixed up with the forest class in the classification process. Thirdly, cropland continued as a dormant loser and dormant gainer throughout the studied three intervals. This is because cropland accounts for the biggest percentage of the landscape composition compared to other LULC categories.

Analysis of intensity at the transition level revealed that cropland gains from forest were higher between 1988 and 1998 compared to the gain in cropland from the similar LULC category over the second study period (1998–2008). However, cropland avoided gains and losses of forest in the third interval (2008–2018). This suggests that the expansion in cropland resulted in a decline in the forest area. Nevertheless, the loss of agricultural area to the forest is associated with the spreading of Mesquite in cropland as we mentioned earlier. Our study also showed the transition of forest to water in the three change periods. This might be linked to misclassification between the two classes as most of the forests in Gedaref state are Nilotic trees such as *Acacia nilotica* and *Acacia seyal*, which lie mainly around the seasonal rivers and watercourses. Similarly, the gains in grassland from the settlement in the first period (1988–1998) could be a likely result of the slight misclassification of wood, grass and hut houses as grassland. The second explanation is that the low resolution of the satellite image for 1988–1998 might have contributed to the misclassification of settlements as grassland. Our intensity analysis also showed the gain of grassland from water in the second and third periods (1998–2008 and 2008–2018). This is explained by the fact that grassland overlaps with water due to the seasonality of water bodies in Gedaref state. Another interesting key finding was observed in grassland losses to water, which was higher in the period between 2008 and 2018, compared to the losses in grassland to the similar LULC category over the first and second periods. This is perhaps as a result of the construction of the Upper Atbara and Saiteet Dam complex and the increase of water storage ponds for drinking water, domestic use and irrigation of agricultural land during this period. The settlement loss targets water and grassland in the three study periods with the addition of the forest in the second period (1998–2008). This could be linked to pastoralism movements through seasonal migration routes and settling where there are grass and water. Whereas, gaining in settlement target cropland and water in the second and third periods, respectively, with no gain from any LULC class in the first period. Settlement is usually located in the flat areas, where cropland can be also found in these areas. Hence, the expansion in settlement areas, is likely to target cropland and water gathering sites.

The predicted results revealed that by 2028 and 2048, cropland is expected to increase by 0.12% and 0.72%, respectively as compared to 2018. This is partly attributed to the expansion of mechanized farming in the study area. Similarly, the settlement might be increased from 0.44 to 0.50% between 2018 and 2048. This could be attributed to the rapid population growth and refugee influx in the study area [90]. Whereas the forest acreage is anticipated to decrease from 0.47% in 2018 to 0.41% in 2048. This could be linked to illegal cutting, overgrazing and mechanized agriculture since the population in Gedaref sate depends mainly on farming activities for livelihoods [44, 91]. The model prediction showed that the trend of grassland will continuously decrease by 7.78% in 2048 from 8.59% in 2018. Water will be relatively consistent in the region in 2018, 2028 and 2048 by 0.89%, 0.89% and 0.88%, respectively.

The findings presented in this study could guide policymakers and different stakeholders to effectively plan and manage the landscape in Gedaref state, Sudan. Also, the study provides some insights on the main drivers that could play a vital role in changing the current and future landscape structure in most important rainfed farming areas in Sudan. Knowing the areal extent, change rate, intensity and transition of important LULC categories like cropland and grassland over 30 years could enable an informed crop and grassland production monitoring. Specifically, the results of the present study could complement the findings of Osman et al. [20], who predicted the relationship between climate factors and crop yield in Gedaref state under climate warming. Hence, total crop production in Gedaref could be predicted and forecasted using both study findings. In general, different land use and environmental policy and planning initiatives in Gedaref state or even in Sudan at large could make use of the findings presented in this study.

Despite the fact that we have used a robust and efficient machine learning RF algorithm in our LULC classification experiment, the method has some limitations. For instance, when a large number of decision trees are used, the algorithm can be too slow to make the classification predictions as it requires more computational power. Another disadvantage of RF is that the method is a black or grey box approach with very little control over what the algorithm does [58]. On the other hand, we simulated the future (2028 and 2048) LULC predictions using the current natural and anthropogenic factors that might considerably change in the future. In addition, other factors that could play a significant role in the future LULC shift like fire, flood, conflict, and other geopolitical and socio-economic variables were not considered in our study. We also mapped and detected the LULC shift till the year 2018 in Gedaref state, which could have slightly changed in the year 2022. But, the LULC changes in such agro-systems might take a longer time period (more than four years) to take place.

## Conclusions

In conclusion, this study is the first attempt to map and predict future LULC change and their intensities and underline the processes that cause change in the landscape of Gedaref state. Our results showed that LULC in Gedaref has undergone a distinct change in 30 years period (1988–2018) with a considerable decline in forest and grassland areas and an expansion in settlement areas. Our classified LULC maps and model validation for future LULC prediction provided high accuracy (overall correctness = 87%). This demonstrates the possibility of mapping and predicting LULC classes using on-screen reference data from Google Earth images, dense multi-date Landsat images, RF classifier and CA-ANN model. Our findings, provide information on LULC patterns in Gedaref region that could be useful for designing management plans and developing policies for assessing and monitoring crop and grassland production, other natural resources produce, landscape fragmentation and degradation, and ecosystem functions. This information is, therefore, critical in managing one of the most important rainfed agricultural landscapes in Sudan.

## Supporting information

S1 Table LULC change transition matrix from 1988–2018: Area (ha) and rate of change per year. (DOCX)

S2 Table LULC change transition matrix from 1998–2018: Area (ha) and rate of change per year. (DOCX)

S3 Table LULC change transition matrix from 2008–2018: Area (ha) and rate of change per year. (DOCX)
