# Supplementary material for: Mapping, intensities and future prediction of land use/land cover dynamics using google earth engine and CA- artificial neural network model
Source: PLoS One. 2023 Jul 24;18(7):e0288694. doi: 10.1371/journal.pone.0288694 (PMC10365312; doi:10.1371/journal.pone.0288694)
Supplement: S1 Table — (DOCX) [file pone.0288694.s001.docx]

**Mapping, intensities and future prediction of Land use/Land cover dynamics using google earth engine and CA- artificial neural network model**

Maysoon A. A. Osman^1, 2, 3*^, Elfatih M. Abdel-Rahman^2^, Joshua Orungo Onono^1,4^, Lydia A. Olaka^1,5^, Muna M. Elhag^6^, Marian Adan^2^ and Henri E. Z. Tonnang^2^

^1^Department of Earth and Climate Sciences, Faculty of Science and Technology, University of Nairobi, P. O. Box 30197, 00100 Nairobi, Kenya

^2^International Centre of Insect Physiology and Ecology (icipe), P.O. Box 30772, Nairobi 00100, Kenya

^3^Department of Forestry and Environment, Faculty of Forest Sciences and Technology, University of Gezira, P.O Box: 20, Wad Madani 21111, Sudan

^4^Department of Public Health, Pharmacology and Toxicology, University of Nairobi, P. O. Box 29053–00625, Nairobi 00100, Kenya

^5^Current address: Department of Geoscience and Environment, School of Physics and the Environment, Technical University of Kenya, P.O. Box 52428 – 00200, Nairobi, Kenya

^6^ Water Management and Irrigation Institute, University of Gezira; P.O. Box 20, Wad Medani 21111, Sudan

* Correspondence: Maysoon A. A. Osman, [mosman@icipe.org](mailto:mosman@icipe.org) ; [osmanmaysoon@gmail.com](mailto:osmanmaysoon@gmail.com)

**Table S1. LULC change transition matrix from 1988–2018: Area (ha) and rate of change per year**

| **LULC 1988** | **LULC 2018** | | | | | | |
| --- | --- | --- | --- | --- | --- | --- | --- |
|  | **LULC Class** | **Cropland** | **Forest** | **Grassland** | **Water** | **Settlement** | **Row total**  **1988** |
|  | **Cropland** | **4769947.53*** | 18499.86 | 197685.27 | 17216.91 | 20608.47 | 5023958 |
|  | **Forest** | 59062.86 | **8104.77** | 1052.37 | 757.89 | 75.24 | 69053.13 |
|  | **Grassland** | 880106.76 | 1289.79 | **348050.7** | 23876.19 | 1156.41 | 1254480 |
|  | **Water** | 14182.74 | 2157.66 | 2086.38 | **15490.8** | 207.27 | 34124.85 |
|  | **Settlement** | 361.53 | 7.29 | 127.89 | 7.83 | **6234.48** | 6739.02 |
|  | **Column total (2018)** | 5723661.42 | 30059.37 | 549002.61 | 57349.62 | 28281.87 | 5723661.42 |
|  | **Class changes** | 953713.89 | 21954.6 | 200951.91 | 41858.82 | 22047.39 | 953713.89 |
|  | **Chang difference**  **(1988 - 2018)** | 699703.38 | -38993.76 | -705477.24 | 23224.77 | 21542.85 | 699703.38 |
|  | **Rate of change/year** | 31790.463 | 731.82 | 6698.397 | 1395.294 | 734.913 | 31790.463 |

*The values along the transition diagonals of the table matrix represent the LULC class from time 1 to time 2 (time 2 > time1), with the area of LULC categories (ha) that remained unchanged through the time period; while the off-diagonal data represent a transition from one LULC class to another.
