# Supplementary material for: Mapping, intensities and future prediction of land use/land cover dynamics using google earth engine and CA- artificial neural network model
Source: PLoS One. 2023 Jul 24;18(7):e0288694. doi: 10.1371/journal.pone.0288694 (PMC10365312; doi:10.1371/journal.pone.0288694)
Supplement: S3 Table — (DOCX) [file pone.0288694.s003.docx]

**Mapping, intensities and future prediction of Land use/Land cover dynamics using google earth engine and CA- artificial neural network model**

Maysoon A. A. Osman^1, 2, 3*^, Elfatih M. Abdel-Rahman^2^, Joshua Orungo Onono^1,4^, Lydia A. Olaka^1,5^, Muna M. Elhag^6^, Marian Adan^2^ and Henri E. Z. Tonnang^2^

^1^Department of Earth and Climate Sciences, Faculty of Science and Technology, University of Nairobi, P. O. Box 30197, 00100 Nairobi, Kenya

^2^International Centre of Insect Physiology and Ecology (icipe), P.O. Box 30772, Nairobi 00100, Kenya

^3^Department of Forestry and Environment, Faculty of Forest Sciences and Technology, University of Gezira, P.O Box: 20, Wad Madani 21111, Sudan

^4^Department of Public Health, Pharmacology and Toxicology, University of Nairobi, P. O. Box 29053–00625, Nairobi 00100, Kenya

^5^Current address: Department of Geoscience and Environment, School of Physics and the Environment, Technical University of Kenya, P.O. Box 52428 – 00200, Nairobi, Kenya

^6^ Water Management and Irrigation Institute, University of Gezira; P.O. Box 20, Wad Medani 21111, Sudan

* Correspondence: Maysoon A. A. Osman, [mosman@icipe.org](mailto:mosman@icipe.org) ; [osmanmaysoon@gmail.com](mailto:osmanmaysoon@gmail.com)

**Table S3. LULC change transition matrix from 2008–2018: Area (ha) and rate of change per year**

| **LULC 1998** | **LULC 2018** | | | | | | |
| --- | --- | --- | --- | --- | --- | --- | --- |
|  | **LULC Class** | **Cropland** | **Forest** | **Grassland** | **Water** | **Settlement** | **Row total**  **1998** |
|  | **Cropland** | **4742922.55*** | 16962.57 | 251640.27 | 16340.4 | 17681.04 | 5289566.13 |
|  | **Forest** | 271130.4 | **90103.5** | 5490.9 | 5274.9 | 201.6 | 37217.16 |
|  | **Grassland** | 692445.96 | 1429.2 | **294029.82** | 23587.2 | 1157.76 | 1012649.94 |
|  | **Water** | 17005.68 | 2655 | 2657.16 | **16888.14** | 149.94 | 39363.12 |
|  | **Settlement** | 157.86 | 2.25 | 126.27 | 6.39 | **9272.97** | 9565.74 |
|  | **Column total (2018)** | 5723662.45 | 30056.44 | 548998.87 | 57366.22 | 28278.13 |  |
|  | **Class changes** | 980739.9 | 21049.02 | 259914.6 | 45208.89 | 19190.34 |  |
|  | **Chang difference**  **(1998 - 2018)** | 434096.32 | -7160.72 | -463651.07 | 18003.1 | 18712.39 |  |
|  | **Rate of change/year** | 49036.995 | 1052.451 | 12995.73 | 2260.4445 | 959.517 |  |

*The values along the transition diagonals of the table matrix represent the LULC class from time 1 to time 2 (time 2 > time1), with the area of LULC categories (ha) that remained unchanged through the time period; while the off-diagonal data represent a transition from one LULC class to another.
